# Supplementary material for: EnzML: multi-label prediction of enzyme classes using InterPro signatures
Source: BMC Bioinformatics. 2012 Apr 25;13:61. doi: 10.1186/1471-2105-13-61 (PMC3483700; doi:10.1186/1471-2105-13-61)
Supplement: Addtional file 5 — The Java code to format the data files, evaluate and predict. The file enzml_java_code.tar.gz contains the Java code used to format database data to ARFF and XML formats, to execute cross and train-test (jackknife) evaluations and to record evaluation results to database. More information is included in the readme.txt file and the Javadoc files. The code can be used with a MySQL database. To use a different database software, other JDBC drivers might be required. [file 1471-2105-13-61-S5.gz › java_code/enzml2011/doc/uk/ac/ed/inf/csb/taweka/test/InstancesFactoryTest.html]

InstancesFactoryTest


---


|  |  |  |
| --- | --- | --- |
| **Overview** \| **Package** \| **Class** \| **Use** \| **Tree** \| **Deprecated** \| **Index** \| **Help** | |  |
| **PREV CLASS**   **NEXT CLASS** | **FRAMES**    **NO FRAMES**     **All Classes** |  |
| SUMMARY: NESTED \| FIELD \| CONSTR \| METHOD | DETAIL: FIELD \| CONSTR \| METHOD |  |


---


## uk.ac.ed.inf.csb.taweka.test Class InstancesFactoryTest

```
java.lang.Object
  junit.framework.Assert
      junit.framework.TestCase
          uk.ac.ed.inf.csb.taweka.test.InstancesFactoryTest
```

**All Implemented Interfaces:**: junit.framework.Test

---

``` public class InstancesFactoryTest extends junit.framework.TestCase ```

Class

**Version:**
:   22 Feb 2008

**Author:**
:   Luna De Ferrari luna.deferrari-at-ed.ac.uk

---

**Constructor Summary**

`InstancesFactoryTest()`


| **Method Summary** | |
| --- | --- |
| `static InstancesFactory` | `instancesFactory()` |
| `void` | `testWriteInstance()`             public void testFillNonExistingInstances() { InstancesFactory factory = InstancesFactoryTest.instancesFactory(); SparseInstance instance = new SparseInstance(factory.getDataSet() .getAttributes().size()); // set the dataset (Instances) for the instance instance.setDataset(factory.getDataSet().getInstances()); // fill instance attribute values instance = factory.fillInstance("empty", instance); assertEquals("@attribute att1 {n,y}", instance.attribute(0).toString()); assertEquals("@attribute att3 {n,y}", instance.attribute(2).toString()); assertEquals(4, instance.classIndex()); assertEquals("NaN", Double.toString(instance.classValue())); assertEquals(4, instance.index(0)); assertEquals(5, instance.numAttributes()); assertEquals(1, instance.numValues()); assertEquals("{4 ?}", instance.toString()); } |

**Methods inherited from class junit.framework.TestCase**

`countTestCases, getName, run, run, runBare, setName, toString`

**Methods inherited from class junit.framework.Assert**

`assertEquals, assertEquals, assertEquals, assertEquals, assertEquals, assertEquals, assertEquals, assertEquals, assertEquals, assertEquals, assertEquals, assertEquals, assertEquals, assertEquals, assertEquals, assertEquals, assertEquals, assertEquals, assertEquals, assertEquals, assertFalse, assertFalse, assertNotNull, assertNotNull, assertNotSame, assertNotSame, assertNull, assertNull, assertSame, assertSame, assertTrue, assertTrue, fail, fail, failNotEquals, failNotSame, failSame, format`

**Methods inherited from class java.lang.Object**

`equals, getClass, hashCode, notify, notifyAll, wait, wait, wait`

**Constructor Detail**

### InstancesFactoryTest

```
public InstancesFactoryTest()
```


**Method Detail**

### testWriteInstance

```
public void testWriteInstance()
```

:   public void testFillNonExistingInstances() { InstancesFactory factory =
    InstancesFactoryTest.instancesFactory(); SparseInstance instance = new
    SparseInstance(factory.getDataSet() .getAttributes().size());
    // set the dataset (Instances) for the instance
    instance.setDataset(factory.getDataSet().getInstances());
    // fill instance attribute values instance =
    factory.fillInstance("empty", instance);
    assertEquals("@attribute att1 {n,y}", instance.attribute(0).toString());
    assertEquals("@attribute att3 {n,y}", instance.attribute(2).toString());
    assertEquals(4, instance.classIndex()); assertEquals("NaN",
    Double.toString(instance.classValue())); assertEquals(4,
    instance.index(0)); assertEquals(5, instance.numAttributes());
    assertEquals(1, instance.numValues()); assertEquals("{4 ?}",
    instance.toString());
    }

---


### instancesFactory

```
public static InstancesFactory instancesFactory()
```


---


|  |  |  |
| --- | --- | --- |
| **Overview** \| **Package** \| **Class** \| **Use** \| **Tree** \| **Deprecated** \| **Index** \| **Help** | |  |
| **PREV CLASS**   **NEXT CLASS** | **FRAMES**    **NO FRAMES**     **All Classes** |  |
| SUMMARY: NESTED \| FIELD \| CONSTR \| METHOD | DETAIL: FIELD \| CONSTR \| METHOD |  |


---
